# Intra-puparial development of the black soldier-fly, *Hermetia illucens*

**DOI:** 10.1093/jis/14.1.83

**Published:** 2014-01-02

**Authors:** Karine Brenda Barros-Cordeiro, Sônia Nair Báo, José Roberto Pujol-Luz

**Affiliations:** 1 Laboratório de Microscopia Eletrônica, Departamento de Biologia Celular, Instituto de Ciências Biológicas,Universidade de Brasília, 70910-900, DF, Brazil; 2 Laboratório de Entomologia Forense, Departamento de Zoologia, Instituto de Ciências Biológicas, Universidade de Brasília, 70910-900, DF, Brazil

**Keywords:** forensic entomology, metamorphosis, morphology, Stratiomyoidea

## Abstract

The intra-puparial development of the black soldier-fly, *Hermetia illucens* (L.) (Diptera: Stratiomyidae), was studied based on 125 pupae under controlled conditions in laboratory. The 6 ^th^ instar larvae were reared until they stopped feeding, and the prepupae were separated according to the reduction in larval length and degree of pigmentation and sclerotization of the cuticle. The pupal stage lasted eight days (192 hours). The process of pupation (larva/pupa apolysis) occurred in the first six hours, extroversion of the head and thoracic appendages took place between the ninth and 21 ^st^ hours, and the pharate appeared 21 hours after completing pupation. After pupariation, four morphological phases of intra-puparial development were observed and described.

## Introduction

The Hermetiinae soldier-flies represent a relatively homogeneous group of Stratiomyidae consisting of five genera, *Chaetohermetia* (2 spp., neotropical); *Chaetosargus* (4 spp., neotropical); *Hermetia* (76 spp., cosmopolitan), *Nothohermetia* (1 sp., Australia), and *Pata-giomyia* (1 sp., neotropical). The genus *Hermetia* Latreille has 76 species, 39 of them occurring in the Neotropical region, 20 with distribution including Brazil. Only four species have known larvae: *Hermetia albitarsis* (Brazil), *H. aurata* (Mexico), *H. concinna* (Mexico), and *H. illucens* (cosmopolitan) ( McFadden 1967 ; Woodley 2001 ).

The black soldier fly, *H. illucens* (L.) (Diptera: Stratiomyidae), is economically important because its larvae feed on and are involved with cycling organic matter ( Lardé 1990 ) and also inhibit and control the oviposition and development of *Musca domestica* in manure management systems ( Sheppard 1983 ; Bradley and Sheppard 1984 ). There are also concerns regarding this species because of its association with cases of enteric myiasis in humans and other animals ( Adler and Brancato 1995 ; Manrique-Saide et al. 1999 ; Calderón-Arguedas et al. 2005 ). In addition, records of the larvae and pupae of *H. illucens* occurring in human carcasses indicate that this species is also important in studies of forensic entomology, and that its development can be used to estimate postmortem interval ( Catts and Haskell 1990 ; Lord et al. 1994 ; Turchetto et al. 2001 ; Tomberlim et al. 2004; Pujol-Luz et al. 2008 ).

Most of the studies that have investigated the events of metamorphosis in Diptera described only the pupariation process, which ends in the formation of the puparium. The morphological changes that occur during intra-puparial development have been extensively studied in Muscoidea and Oestroidea (e.g., Wolfe 1954 ; Bennett 1962 ; Fraenkel and Bhaskaran 1973 ; Lello et al. 1985 ; Scholl and Weintraub 1988 ; Cepeda-Papacios and Scholl 2000 ; Colwell et al. 2006 ), while intra-puparial development in Stratiomyioidea has never been investigated. In this paper we describe some events in *H. illucens* development, including the chronology and morphological changes observed during intra-puparial development, under controlled laboratory conditions.

## Materials and Methods

Two hundred 6 instar larvae (L6) of *H. illucens* were reared and observed in a BOD incubator chamber (27 ± 1.0°C, 60 ± 10% RH, 12:12 L:D) until they ceased feeding, which marks the onset of the pupariation process. The prepupae were separated and placed in plastic containers with vermiculite. During the first 48 hours, five pupae were fixed every three hours; after this period, during the next six days, this process was repeated every 16 hours until the emergence of the adults. A total of 125 pupae were dissected in the experiment. All specimens were fixed in Car-noy’s solution (48 hours), then in formic acid (5%) for another 48 hours; after that, they were transferred for permanent preservation in ethanol 70. The adults that emerged (n = 75) were fixed at low temperature (-20°C) and maintained in ethanol (70%) with the pupae. The minimum time intervals for each event (prepupae, pupae) were recorded. Ten pupae were cleaned in hot lactic acid; this process permits the observation of respiratory system structures that are useful. The specimens were dissected and photographed with Leica DM2000® ( www.leica-microsystems.com ) and Zeiss AxioCam®MRc ( www.zeiss.com ) microscopes.

The terminology and concepts used to describe the processes of pupariation and pupation and the puparium morphology were adapted from Fraenkel and Bhaskaran (1973) , Costa and Vanin (1985) , Cepeda-Palacios and Scholl (2000) , and are defined as follows:

(i) Pupariation: period between the time that the larvae cease feeding to complete immobilization and reduction in length of the larvae. A reduction in their mobility and a retraction of the segments gradually occur. The cuticle becomes progressively more opaque, pigmented and sclerotized.

(ii) Pupation or intrapupal development: all events that occur from larval-pupal apolysis until the emergence of the adult fly.

(iii) Larva-pupa apolysis: once the pupariation process has finished, larval-pupal apolysis takes place, resulting in the formation of the adult epidermis and its separation from the last larval skin, which will form the puparium.

(iv) Cryptocephalic pupa: a phase also known as hidden head; in this phase it is impossible to distinguish the head and the thoracic appendages externally; the imaginal discs of the appendages and the cephalic vesicle (cerebral and cephalic ganglia) are located below the thoracic and abdominal segment.

(v) Phanerocephalic: in this phase there is the extroversion of the cephalic capsule and the thoracic appendages. This phase also marks the beginning of the apolysis process between pupa and adult.

(vi) Pharate adult: the longest phase of intrapupal development, corresponding to the maturation of the adult.

(vii) Imago: the final form of the insect after metamorphosis.

## Results

### Pupariation

**Figure 1.**
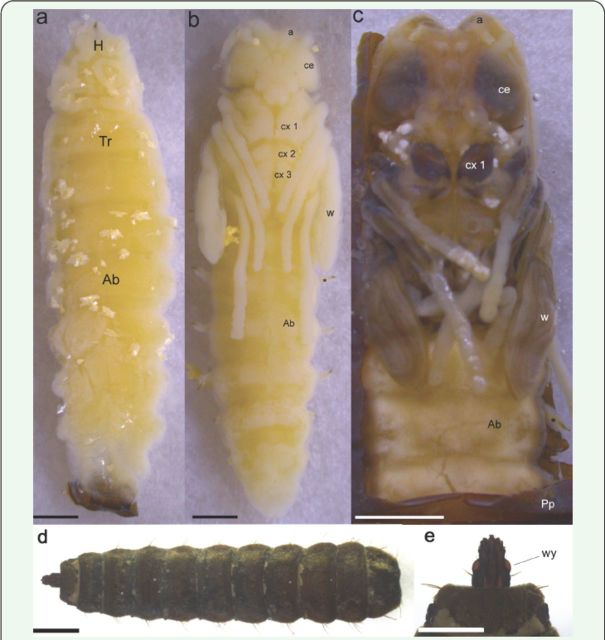
Sequence of the intra-puparial development of *Hermetia illucens* (ventral view). (a) Cryptocephalic pupa; (b) pharate adult; (c) imago; (d) pupae; (e) detail of the head (dorsal view). Abbreviations: Ab, abdomen; a, antenna; ce, compound eyes; cx, coxa; H, head; Pp, puparium; Tr: thorax; w, wing; wy, white-eyes; * respiratory tubes. Scale bars: 1.6 mm (a,b), 2.5 mm (c) 1.2 mm (d), and 0.7 mm (e). High quality figures are available online.

The larvae of *H. illucens* remained in the vermiculite substrate between two and 15 days and buried in the substrate in scotophase, when there was a reduction in their mobility. The pupa was 1/3 the size of the puparium and, because of the reduction in the tissue in the anterior part of the puparium, there was a change in eye color from reddish to white or transparent; the abdomen also folded 45° to the ventral region, and the cuticle gradually became opaque and sclerotized ( Figure 1d , e).

### Larval-pupal apolysi

The process was observed in a dorsal-ventral direction and from the end of the abdomen to the head. The apolysis duration was 4.8 ± 1.1 hours, being completed in a minimum time of six hours ( Table 1 ).

**Table 1.**
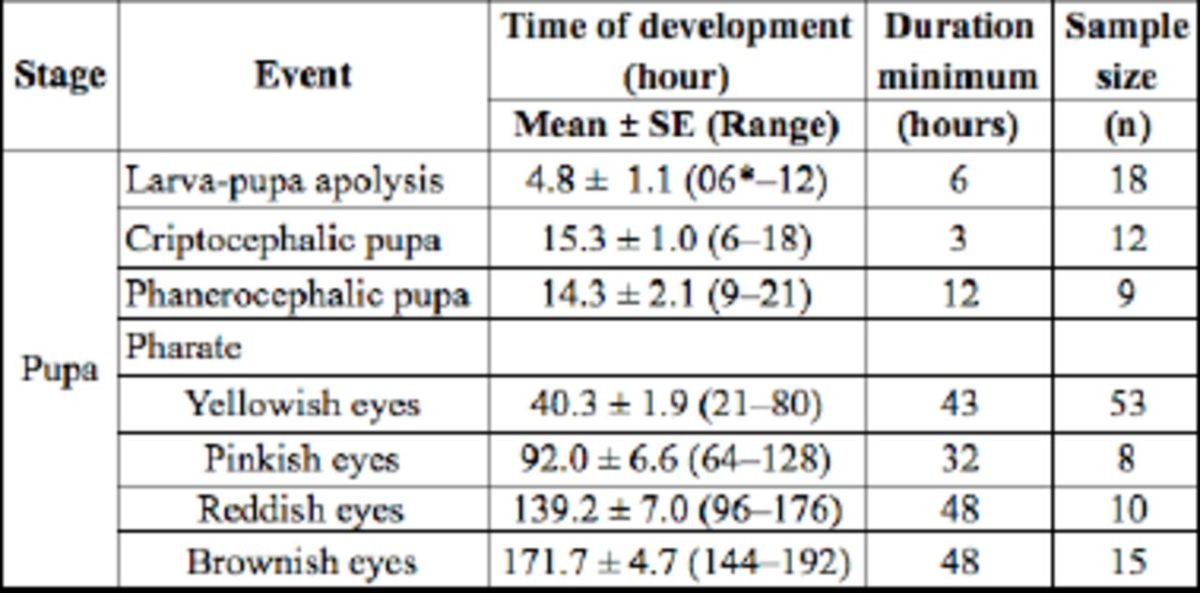
Intra-puparial development of *Hermetia illucens.*

*The apolysis was complete

### Cryptocephalic pupa ( Figure 1a )

Formation of a hardened, opaque, and pigmented puparium; this phase retained almost all the features of L6. The mandibular-maxillary complex was separated from the larva and the pupa and stayed attached to the puparium internal wall. The duration of this event was 15.3 ± 1.0 hours, being completed in a minimum time of three hours, and ended with start of the extroversion of the head and thoracic appendages (Table I).

### Phanerocephalic pupa

Characterized by the extroversion and distinctness of the head, thorax, and abdomen of the pharate adult ( Figure 1b ). This process marked the pupa-adult apolysis, with a duration of 14.3 ± 2.1 hours, being completed in a minimum time of 12 hours ( Table 1 ).

### Pharate adult ( Figure 1b )

**Figure 2.**
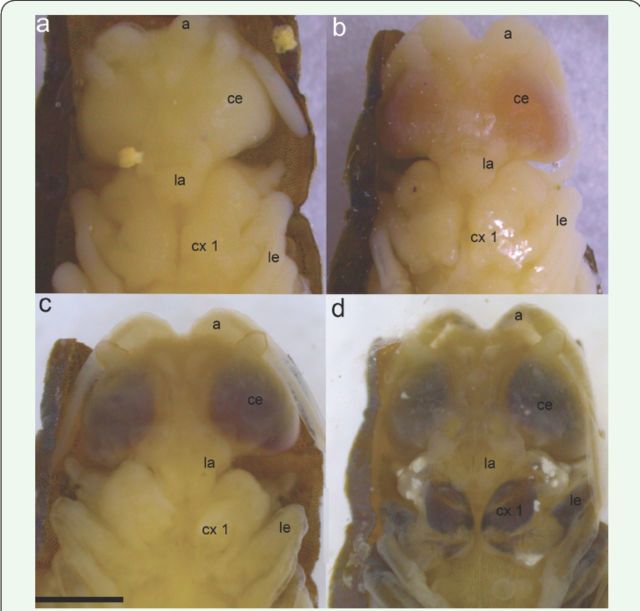
The pharate of *Hermetia illucens* , according to the color of the compound eyes. (a) Yellowish eyes; (b) Pinkish eyes; (c) Reddish eyes; (d) Brownish eyes. Abbreviations: a, antenna; ce, compound eyes; cx, coxa; la, labrum; le, legs. Scale bar: 1.5 mm. High quality figures are available online.

The longest phase of intra-puparial development, which can be divided into four stages according to the color of the eyes; it represents maturation of the adult (adapted from Cepeda-Palacios and Scholl 2000 ): (i) yellowish eyes, 21 ^st^ –64 ^th^ hour, with duration this period being 40.3 ± 1.9 hours ( Figure 2a ; Table 1 ), followed by the definition of head, thorax, abdomen, legs and wings; (ii) pinkish eyes, 64 ^th^ –96 ^th^ hour, a duration of 92.0 ± 6.6 hours ( Figure 2b ; Table 1 ), and the sutures of the thorax and abdomen in dorsal view were observed; (iii) reddish eyes, 96 ^th^ –144 ^th^ hour, a duration of 139.2 ± 7.0 hours ( Figure 2c ; Table 1 ), the T-shaped dorsal thoracic suture in the puparium was observed, and the pharate showed a well-developed antennae and the beginning of the pigmentation of hair, bristles, legs, and wing veins; (iv) dark brownish eyes, 144 ^th^ –192 ^nd^ hours, a duration of 171.7 ± 4.7 hours ( Figure 2d ; Table 1 ), the body of adult was totally formed and fully pigmented.

### Imago and emergence of the adults

The completely formed imagoes ( Figure 1c ) were observed in the 144 ^th^ hour, and the adults emerged from the 192 ^nd^ hour ( Table 1 ).

### Respiratory system

**Figure 3.**
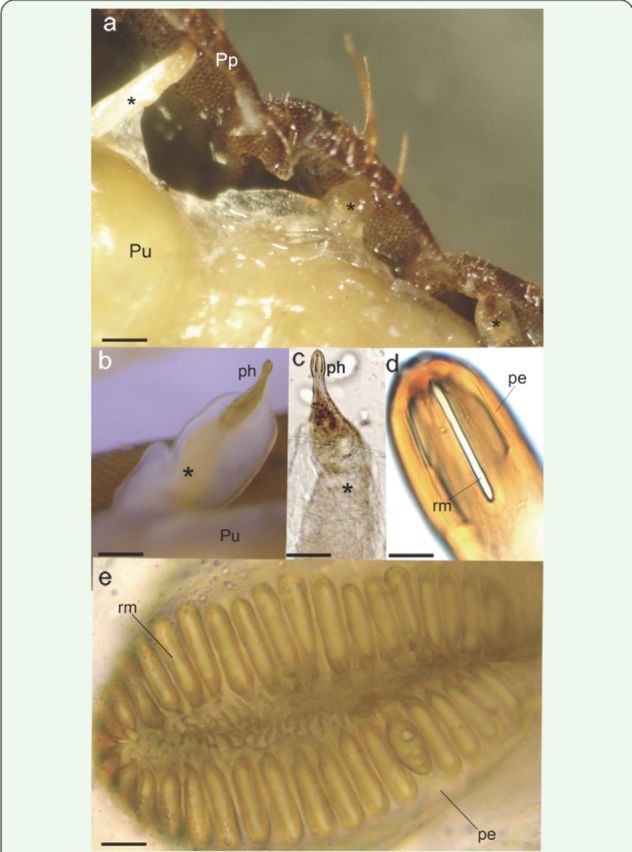
Morphology of the respiratory system of *Hermetia illucens.* (a) Lateral projections of respiratory tubes; (b, c) pupal-horn; (d) tip of the pupal-horn; (e) crown-like respiratory opening. Abbreviations: pe, peritreme; ph, pupal respiratory horn; Pp, puparium; Pu, pupa; rm, rima; * respiratory tubes. Scale bars: 0.3 mm (a), 2.5 mm (b), 3.4 mm (c), 0.6 mm (d), and 1.6 mm (e). High quality figures are available online.

In the pupal stage, the external breathing tubes (pupal-horn) appeared ( Figure 3a –c). A pair of developed internal tubes was present in the first thoracic segment, and another five pairs were distributed in the abdomen segments 2 to 7. Inside the puparium, the tubes became narrowed at the distal end and were prolonged beyond the outer edge, where the ends ( Figure 3a ) presented two different forms: the first was horn-like (Figures 3b–d), 2 ^nd^ to 5 ^th^ abdominal segment, and the second had a crown-like shape ( Figure 3e ), 6 ^th^ and 7 ^th^ abdominal segments.

## Discussion

There are a few morphological studies relating to intra-puparial development, and in some of them ( Irwins-Smith 1920 ; May 1961 ; Rozkošný 1982) the subject was treated without the necessary level of detail. Most authors use the puparia to describe the last instar larvae of Stratiomyidae ( Rozkošný and Kovac 1998 , 2001; Pujol-Luz and Leite 2001 ; Xerez and Pujol-Luz 2001 ; Xerez et al. 2002 , 2003). In Stratiomyidae and Xylomyidae, the pupa is formed within the last larval skin, which is used as a hard cocoon (puparia) impregnated with plates or calcium carbonate crystals (Ca-CO _3_ ) ( Woodley 1989 ). The total time of post-embryonic development in soldier-flies (larva to adult) is variable, lasting for a few weeks to several months in Atlantic Rain Forest and Cerrado biomes (J.R. Pujol-Luz, personal observation). In controlled laboratory conditions, the total duration of the period between the pupal stage and adult emergence of *H. illucens* in this work lasted 192 hours, and during this time we identified four distinct phases or stages: (i) larval-pupal apolysis, (ii) cryptocephalic pupae, (iii) phanerocephalic pupae, and (iv) pharate adult ( Table 1 ).

Apart from the morphological changes associated with the change in eye color, we highlighted some modifications in the structure and pattern of the respiratory system. The respiratory system ( Figure 3 ) of the pupa of *H. illucens* is usually described as amphipneustic, despite the presence of the vestigial breathing tubes in the 2 ^nd^ to 5 ^th^ abdominal segment ( Rozkošný 1982 ; Rozkošný and Kovac 2001 ). However, we can provide a different interpretation based on the findings of this study. The tissular projection of the tracheal system in pharate adults forms a tube that is in contact with the internal pupal wall ( Figure 3a ), and that has slits opening out at the extremity of the pupal horn (Figures 3b–e), suggesting that the spiracles are indeed functional. Thus, the respiratory system should be considered hemipneustic.
